# Characterization of Ethylene–propylene Composites Filled with Perlite and Vermiculite Minerals: Mechanical, Barrier, and Flammability Properties

**DOI:** 10.3390/ma13030585

**Published:** 2020-01-27

**Authors:** Bolesław Szadkowski, Anna Marzec, Przemysław Rybiński, Witold Żukowski, Marian Zaborski

**Affiliations:** 1 Institute of Polymer and Dye Technology, Faculty of Chemistry, Lodz University of Technology, Stefanowskiego 12/16, 90-924 Lodz, Poland; marian.zaborski@p.lodz.pl; 2 Institute of Chemistry, The Jan Kochanowski University, Żeromskiego 5, 25-369 Kielce, Poland; przemyslaw.rybinski@ujk.edu.pl; 3 Department of Chemical Engineering and Technology, Cracow University of Technology, 31-155 Kraków, Poland; pczukows@pk.edu.pl

**Keywords:** ethylene–propylene rubber, perlite, vermiculite, ionic liquids, composite properties

## Abstract

Perlite and vermiculite are naturally occurring minerals, commonly used by industry to obtain highly thermoisolative and/or non-flammable materials. However, there has been little research into the preparation and application of rubber compounds containing these inexpensive mineral fillers. Here, we show the benefits of perlite and vermiculite minerals as fillers for ethylene-propylene rubber (EPM) composites. To obtain more uniform dispersion and improved compatibility between the minerals and the elastomer matrix, 1-allyl-3-methylimidazolium bis(trifluoromethylsulfonyl)imide (AMIMTFSI) and 1-butyl-3-methylimidazolium bis(trifluoromethylsulfonyl)imide (BMIMTFSI) imidazolium ionic liquids (ILs) were added. The mineral fillers were found to be attractive semi-reinforcing fillers, which also act as flame retardants in the elastomer composites. Furthermore, a higher content of vermiculite mineral significantly reduced the air permeability of the composites. The incorporation of ionic liquids into the EPM-filled systems had a considerable effect on the torque increment, crosslink density, and more importantly the flammability of the studied compounds. The application of 2.5 parts per hundred parts of rubber (phr) BMIMTFSI, in particular, reduced the flammability of the EPM composite, as the maximum heat release rate (HRR_max_) decreased from 189.7 kW/m^2^ to 170.2 kW/m^2^.

## 1. Introduction

Elastomer materials have found a wide range of applications in many sectors, such as the aircraft, marine, and construction industries, due to their properties, such as light weight, good toughness, and thermal resistance [1]. Superior qualities such as gas and solvent barrier properties, mechanical strength, and high fire retardancy are additional important characteristics. The fire retardancy of polymer materials can be improved by the addition of flame retardant additives, or by processing following the polymerization process [2]. Recently, carbon nanotubes (CNTs) [3,4,5], layered double hydroxides [6,7], montmorillonite (MMT) [8,9,10], and halloysite [11] have been proposed as halogen-free fire-retardant materials. Other compounds used as eco-friendly fire-retardant compounds in polymers include chitosan phosphate, starch-based materials, and melamine [12,13]. Metal hydroxides and phosphorous compounds are also interesting halogen-free fire-retardant compounds for polymers, due to their low toxicity and low cost [14].

Vermiculite clay ((Mg,Fe,Al)_3_(Al,Si)_4_O_10_(OH)_2_·4H_2_O), a mica-type silicate, is known for its flame-retardant properties [15,16]. An interesting property of vermiculite (VMT) is its exfoliation at elevated temperature, due to the loss of water in the interlayers [17]. Its many advantages include high resistance to chemicals and heat, cation interchangeability, temperature retention, and water adsorption capacity [18,19]. Vermiculite has also been used as a reinforcement for the preparation of polymer nanocomposites. Takahashi et al. [20] used VMT to enhance the gas barrier properties of butyl rubber coatings. Due to its heat-resistant characteristics, VMT has been applied in flame-resistant polypropylene composites [21,22]. When incorporated into polyurethanes, VMT simultaneously improves its tensile and flexural strength [23].

Another interesting inorganic compound is perlite (PT). Formed by the cooling of volcanic eruptions, it is mainly composed of SiO_2_, Al_2_O_3_, K_2_O, Na_2_O, and water. However, depending on its origin PT may also contain TiO_2_, CaO, and MgO [24]. As with VMT, when subjected to thermal treatment PT particles expand up to 20 times in volume, due to the water vaporization [25]. Applications of polyethylene prepared with the addition of PT have been reported previously [26,27]. Recently, Karaca et al. [28] showed that PT enhanced the thermal stability and sound absorption coefficients of polyesters. However, there have until now been no reports in the literature regarding the application of VMT and PT fillers in ethylene–propylene rubber (EPM) elastomer to improve its fire retardancy.

In this work, natural PT and VMT materials were added for the first time to EPM rubber at different concentrations and studied for their effects on its mechanical and flame-resistant properties. Two ionic liquids (ILs), 1-allyl-3-methylimidazolium bis(trifluoromethylsulfonyl)imide (AMIMTFSI) and 1-butyl-3-methylimidazolium bis(trifluoromethylsulfonyl)imide (BIMTFSI), were used as dispersing agents. Their impact on the flammability of the EPM composites was also studied. The ionic salts were selected based on our previous studies, in which they had been used to improve silica and hydrotalcite dispersion in acrylonitrile-butadiene (NBR) and carboxylated acrylonitrile-butadiene (XNBR) rubber, respectively [29,30,31].

## 2. Materials and Methods

### 2.1. Raw Materials

Ethylene–propylene rubber with the commercial name Dutral CO-54, provided by Versalis S.p.A. (Milan, Italy), was applied as an elastomer matrix. The crosslinking system consisted of dicumyl peroxide (DCP) as a curing agent and 1,3,5-triallyl-1,3,5-triazine-2,4,6(1H,3H,5H)-trione (TTT) as a crosslinking co-agent, both purchased from Sigma–Aldrich (Schnelldorf, Germany). 1-Allyl-3-methylimidazolium bis(trifluoromethylsulfonyl)imide (AMIMTFSI) and 1-butyl-3-methylimidazolium bis(trifluoromethylsulfonyl)imide (BMIMTFSI) were also purchased from Sigma–Aldrich. Two different mineral fillers were used, perlite (PT) and vermiculite (VMT) from Torimex-chemicals Ltd. Sp. z o.o. (Lodz, Poland).

### 2.2. Preparation and Characterization Techniques

Elastomer blends were prepared on a laboratory mixing mill with a roll length of 330 mm and a diameter of 140 mm. Each compound was mixed with friction of 1.1, at a temperature of 40 °C, for approximately 15 min. The EPM was first masticated for 3 min on the mill. The other the ingredients (filler, dispersing agents and curing agent) were then successively added to the EPM matrix. Prior the composite preparation, mineral fillers were subjected to the grinding process in classic line planetary ball mill (Fritsch, Idar-Oberstein, Germany). The recipe for the EPM rubber composites is presented in Table 1.

**Figure 1:**
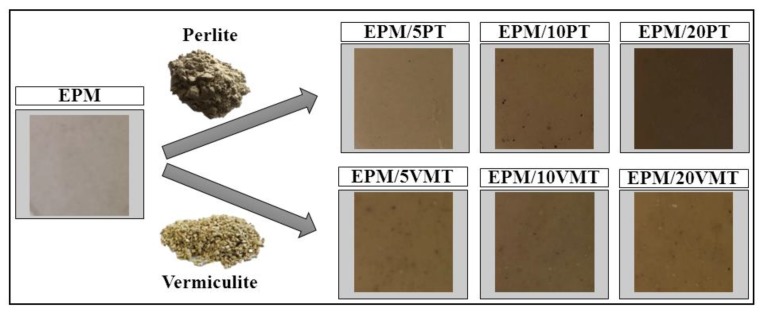
Digital photographs of EPM vulcanizates filled with different contents of perlite and vermiculite.

After 24 h, the elastomer mixtures were subjected to rheometric measurements using a moving die rheometer model (Alpha Technologies, New York, NY, USA), according to the ISO 6502 standard. The curing process was performed on an electrically heated hydraulic press at 160 °C with 15 MPa of pressure for a curing time consistent with the vulcanization parameters. As a result, a series of cured ethylene–propylene rubber composites loaded with different quantities of perlite and vermiculite minerals were obtained (Figure 1).

The crosslink density of the EPM vulcanizates was determined based on the equilibrium swelling method, performed in toluene solvent at room temperature. The crosslink density values were calculated from the Flory–Rehner equation [32]:

υ_e_ = −[ln(1 − V_r_) + V_r_ + µ∙Vr^2^]/[V_0_(V_r_^(1/3)^ − (V_r_/2)] (1)

where υ_e_ is the crosslink density, V_r_ is the volume fraction of the elastomer in swollen gel, V_0_ is the molar volume of the solvent [mol/cm^3^] and µ is the Huggins parameter, determined based on the equation:

µ = μ_0_ + β·V_r_ (2)

where the polymer–solvent interaction parameters for the studied system are μ_0_ = 0.501 and β = 0.273.

The mechanical properties were investigated using a universal strength machine (Zwick, Ulm, Germany) equipped with an extensometer. A tensile test was carried out for five dumbbell-shaped specimens of each composite at a crosshead speed of 500 mm/min, according to the ISO37 standard. The tear resistance of the composites was measured in accordance with the ISO 34-1 standard for three trouser-shaped samples of each composite at a test speed of 50 mm/min. The air permeability of the EPM composites was determined using the manometric method. The gas transmission rate (GTR) was calculated from the equation [33].

(3) $$\mathrm{GTR}=\frac{V_{C}}{R\cdot T\cdot P_{U}\cdot A}\cdot \frac{dp}{dt}$$

where *V_C_* is the volume of the low-pressure chamber (L), *R* is the gas constant 8.31 × 10^3^ ((L∙Pa)/(K∙mol)), *T* is the temperature (K), *P_U_* is the pressure of the gas in the high-pressure chamber (Pa), *A* is the area permeation of the gas through the sample (m^2^), and *dp*/*dt* is the change in pressure over time (Pa/s). The coefficient of gas permeability (*P*) was then calculated as

(4) P=GTR·d

where *d* is the thickness of the specimen (m). The morphology of the mineral fillers and composites was observed via scanning electron microscopy (SEM). Images were taken on a LEO 1530 Gemini scanning electron microscope (Zeiss/LEO, Oberkochen, Germany). Prior to the measurements, the vulcanizates were broken in liquid nitrogen. A cone calorimeter (Fire Testing Technology Ltd., East Grinstead, UK) was applied to evaluate the flammability of the EPM composites, according to the PN-ISO 5660 standard. Each squared specimen, of dimensions 100 mm × 100 mm × 2 mm, was irradiated horizontally using a 35 kW/m^2^ incident heat flux. Thermogravimetric analysis (TGA) was performed using a thermal analyzer (Jupiter STA 449F3, Netzsch Company, Selb, Germany) in a temperatures range of 25–700 °C with a heating rate of 10 °C min^−1^ in a nitrogen atmosphere.

## 3. Results

### 3.1. Curing Measurements

The primary aim of this research was to investigate the effect of mineral fillers, PT and VMT, on the curing kinetics of EPM rubber. The cure properties were studied at 160 °C for 30 min using a moving die rheometer (MDR). The results of rheometric tests showed that the addition of PT and VMT had a significant influence on the curing behavior of EPM composites. The kinetic parameters of the EPM composites are summarized in Table 2.

First, it can be noted that the application of the mineral fillers caused an increase in the minimum torque moment of the EPM mixtures. The minimum torque value was related to the viscosity of the composites. The enhanced M_min_ following the addition of filler could indicate a hydrodynamic effect in the rubber systems filled with the rigid filler particles [34]. Furthermore, because of the relative compatibility between the rubber molecular chains and the mineral, ∆M increases with the increasing loading of PT. The increment of torque during vulcanization is an indirect measurement of the degree of elastomer crosslinking. Hence, it can be concluded that the incorporation of PT will result in the enhancement in the crosslink density of EPM/PT compounds. On the other hand, no significant changes in ∆M were observed for the EPM/VMT blends. For both fillers, a slight reduction in optimum curing time and scorch time was observed, in comparison with the neat EPM. The early start of the crosslinking process, as indicated by the curing times, is evidence of the positive contribution made by the studied minerals to the curing process.

The crosslink density values (see Table 2) corresponded with the variation in torque differences. The crosslink density of the EPM compounds increased in proportion to the content of the mineral fillers, with the maximum reached at 20 phr (5.9 mol/cm^3^) of PT loading. The only exception was vulcanizate filled with 20 phr of VMT, which showed a lower crosslink density value in comparison to the reference sample. These results suggest that the application of PT or VMT as fillers in EPM rubber may also have substantial impact on their mechanical performance. The results from Table 2 further show that the application of ionic liquids contributed to a slight reduction in the optimum curing time and scorch time of the studied composites. This is in agreement with data in the literature [35], which suggests that ionic liquids may act as catalysts for interface reactions and thereby significantly affect rheometric parameters. In our study, the crosslink density values of the EPM/VMT compounds were found to increase by approximately 2 mol/cm^3^. There was, however, no clear effect on the IL structure. Furthermore, the EPM/VMT mixtures exhibited an enhanced increment of torque following the incorporation of TFSI-based (bis(trifluoromethylsulfonyl)imide) imidazolium ILs, regardless of the cation type. The improvement in the kinetic parameters of the systems with ILs can be also explained by the more homogenous distribution of the additives in the polymer matrix, which led to better contact between the components of the vulcanization system.

### 3.2. Mechanical Properties

**Figure 2:**
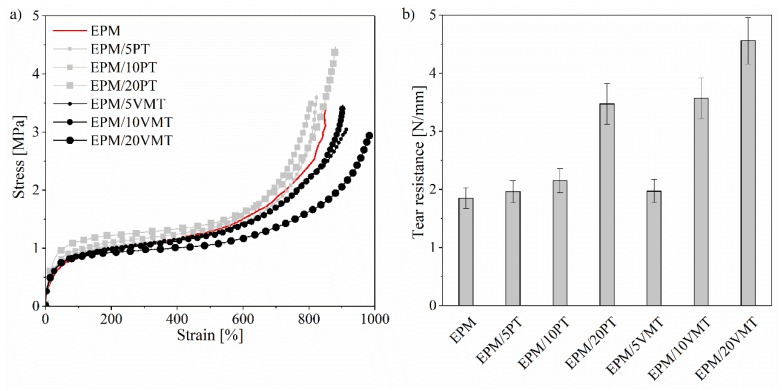
Mechanical curves of the studied EPM composites: (**a**) tensile test; (**b**) tear resistance.

**Figure 3:**
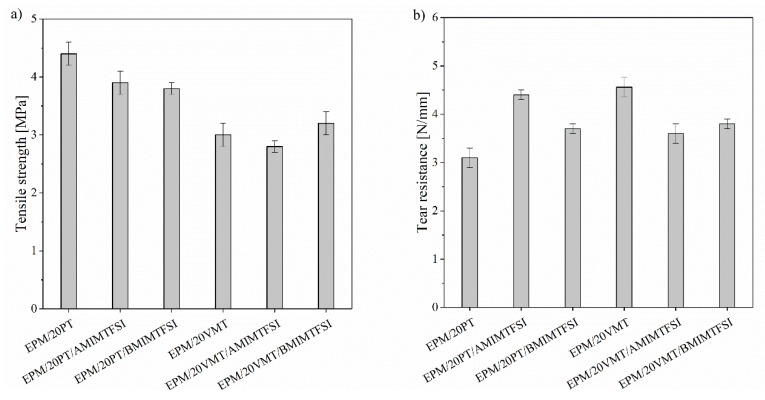
Mechanical parameters of the EPM composites with 20 phr (parts per hundred parts of rubber) of filler and 2.5 phr of ionic liquids: (**a**) tensile strength; (**b**) tear resistance.

The mechanical properties (tensile strength and tear resistance) of the EPM-filled composites are presented in Figure 2. Perlite was found to have a low reinforcing effect, as the tensile strength of the EPM/PT systems increased slightly from 3.4 MPa for neat EPM to 4.5 MPa for the composite filled with 20 phr of PT mineral. However, the tear resistance of the EPM/PT composites increased rapidly with increasing contents of PT in the sample. These improvements in the mechanical properties of EPM/PT materials can be explained by their enhanced crosslink density and the relatively uniform dispersion of the PT particles. In the case of EPM/VMT composites, the tensile strength values remained unchanged or slightly decreased in comparison with the reference sample. The opposite results were obtained by Tjong et al. [36], after incorporation of VMT into a polypropylene (PP) matrix. In their study, the modification of VMT with maleic anhydride probably improved its compatibility with the PP matrix. On the other hand, VMT had a very strong impact on the tear resistance of the EPM compounds, which increased considerably from 1.90 N/mm up to 4.56 N/mm for the EPM/20VMT sample. Composites with layered fillers have been also been reported to have high tear resistance in other previous works [37,38]. The various effects of the studied minerals on the mechanical performance of EPM composites can be explained by the differences in the morphological features of the fillers and their compatibility with the EPM matrix. Applying TFSI-based imidazolium salts did not cause marked alterations in their mechanical parameters. The tensile strengths of the samples containing ILs were slightly lower, which is most likely to be related to the plasticizing effect of the studied ILs [39] (Figure 3a). However, the applied ILs may have a positive effect on the tear resistance of the perlite-filled EPM composites. It can be observed that the tear resistance of the EPM/20PT composite increased in both cases following the application of AMIMTFSI and BMIMTFSI, by approximately 42% and 20%, respectively (Figure 3b).

### 3.3. Barrier Performance

**Figure 4:**
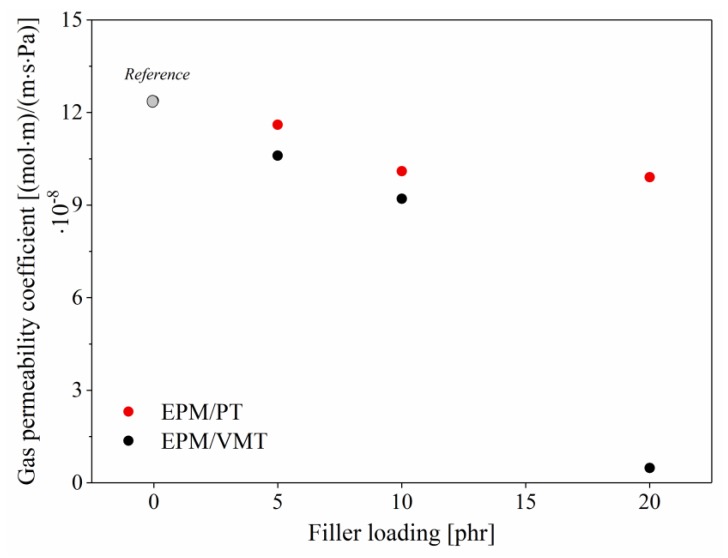
Gas permeability coefficient values for EPM composites filled with different contents of perlite and vermiculite.

The next objective of this study was to explore the gas permeation characteristics of EPM composites filled with PT and VMT. It is known from the literature [40,41] that many different mineral fillers, such as montmorillonite or saponite, can be employed to improve the barrier performance of various polymeric materials. Therefore, EPM composites filled with increasing contents of PT and VMT were investigated in terms of their gas permeability, using the manometric method. The results are presented in Figure 4.

Figure 4 shows that the incorporation of VMT filler significantly improved the barrier performance of the EPM matrix. This is evidenced by the sharply reduced permeability coefficients of the EPM/VMT composites in relation to the reference. The barrier properties of the EPM systems improved with increasing concentrations of the VMT filler. The most visible improvement was observed for 20 phr of VMT, when the gas permeability coefficient was reduced by approximately 12 × 10^8^ ((mol∙m)/(m∙s∙Pa)) compared to neat EPM. This can be explained by the presence of an impermeable clay mineral layer formed by the plate-like VMT particles or by the restricted motion of rubber chains in the vicinity of the mineral filler particles [42]. Gas molecules must travel around individually deposited VMT platelets or stacks, which significantly extends the diffusion length traveled. Different results were observed for EPM composites containing perlite filler. In this case, the gas permeability coefficient values were similar to the reference, regardless of the filler concentration. The different geometric structures of the fillers (spherical for PT, lamellar for VMT) could have a decisive impact on the barrier properties of the EPM-filled composites. In addition, the effect of studied ILs on the barrier performance of the EPM composites were analyzed. The obtained results revealed that incorporation of 2.5 phr of TFSI-based imidazolium ILs into EPM did not affect the air permeability of the investigated elastomer composites.

### 3.4. Thermal Stability

**Figure 5:**
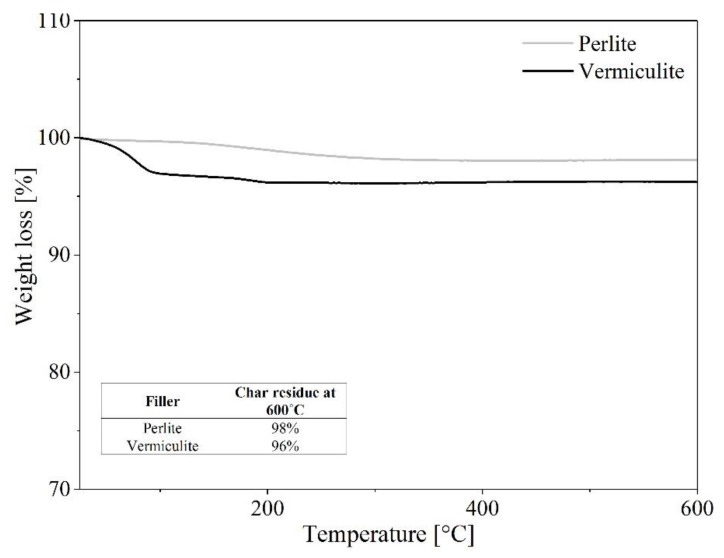
Thermogravimetric curves of the perlite and vermiculite minerals.

**Figure 6:**
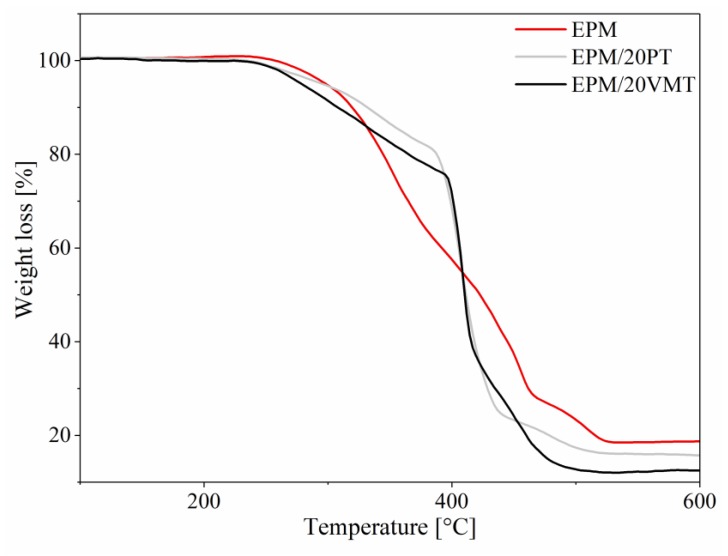
Thermogravimetric curves of the unfilled EPM elastomer and EPM composites with 20 phr of perlite and vermiculite fillers.

The thermal stability of the studied mineral fillers as well as of the EPM composites was evaluated based on thermogravimetric analysis (TGA). The TGA curves and corresponding data are presented in Figure 5 and Figure 6 and Table 3.

As shown in Figure 5, the thermal stability of both studied fillers is very high. The char residues registered at 600 °C for perlite and vermiculite were 98% and 96%, respectively. The TGA curve of the vermiculite filler revealed slight weight loss at a temperature of approximately 100 °C, which is typical for this mineral [43] and corresponds to physisorbed water molecules. These results suggest that the studied minerals are resistant to increasing heat in the examined temperature range. Thus, it can be supposed that the incorporation of perlite and/or vermiculite mineral into EPM may affect the thermal properties of the resulting composites, by protecting the elastomer matrix against high temperatures.

From Figure 6, it can be seen that all the EPM composites are stable, up to approximately 250 °C. The weight loss curves indicate that the thermal degradation of the EPM rubber compounds consisted of three loss steps. The first relatively slow mass loss occurred at temperatures between 200 and 400 °C, the second step can be observed in the range of 400–470 °C, the third step was found to be between 470–530 °C. It should be noted that at the beginning of the second step of thermal degradation, the weight of the EPM/20PT and EPM/20VMT samples were 80% and 74%, respectively, while for neat EPM rubber the weight was as much as 55%. The weight loss values determined for these samples suggest that the addition of both PT and VMT to the studied EPM composites clearly limits its rate of thermal decomposition in the temperature range of ΔT = 200–400 °C. However, above 440 °C the thermal decomposition of both the EPM/20PT and EPM/20VMT samples occurs with much more intensity than in the case of unfilled EPM vulcanizate, which means that the studied minerals may negatively affect the efficiency of thermal crosslinking and cyclization. For this reason, the char residue (P_600_) assumes a higher value for crosslinked EPM rubber in comparison with its filled composites.

### 3.5. Flammability (Cone Calorimetry)

**Figure 7:**
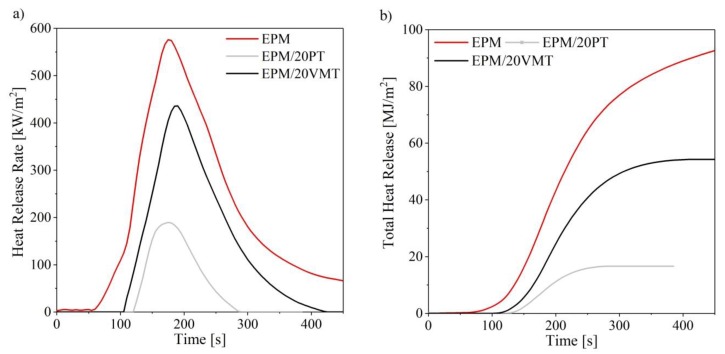
Cone calorimetry test results showing flammability parameters of the EPM composites: (**a**) HRR vs. time and, (**b**) THR vs. time curves.

The cone calorimetry test (CCT) is well-known as an effective bench-scale tool for investigating the flammability of polymer composites. This technique provides data that correlate well with those obtained from full-scale experiments [44]. Therefore, CCT was employed to evaluate the PT and VMT minerals as potential flame retardants for use in EPM rubber composites. The CCT experiments were performed for EPM composites with increasing loading of the fillers. The results are presented in Table 4 and Figure 7.

From the CCT data, it is clear that the flammability parameters were significantly reduced by the incorporation of the studied minerals into the polymer matrix. For instance, in the case of EPM/20PT, the HRR_max_ parameter was reduced by 67% and the THR parameter by 76% with respect to the reference sample, while in the case of EPM/20VMT they fell by 25% and 42%, respectively. Furthermore, the effective heat of combustion (EHC) parameter also decreased significantly with increasing loading of the fillers, suggesting lower flammability. This decrease in EHC may also be connected with the release of physisorbed water from the minerals during thermal decomposition, as the EHC parameter measured in the CCT experiment corresponds closely to the flame burning condition and thus to the combustion of volatiles from the material [45]. Importantly, the EPM-filled composites containing TFSI-based imidazolium ILs exhibited the sharpest reduction in terms of flammability. This means that the studied thermally-stable ionic liquids improved the flame retardancy of the EPM/PT and EPM/VMT systems.

It has been previously reported that TFSI-based ionic liquid may affect the thermal stability of some rubber composites [46]. Generally, the improvement in the flame retardancy of the EPM composites filled with PT and/or VMT minerals may be explained by the fact that the minerals inhibit the degradation rate of EPM rubber in the initial stage of the thermal degradation process. Moreover, due to the very the high thermal insulation and fire resistance of these minerals they may hinder heat transfer within the composite matrix. On the other hand, after the burning process, the filler particles may form so-called filler islets in the polymer matrix, which do not provide sufficient protection against external heat sources. The fillers cannot therefore be said to have a positive effect on the structure and/or insulating properties of the boundary layer. However, PT and VMT minerals can be considered as inexpensive and natural flame-retardant agents for elastomer composites, which are, above all, effective at relatively low concentrations, as evidenced by the considerably reduced HRR, HRR_max_, THR, and EHC parameters (Table 4).

### 3.6. Morphology

**Figure 8:**
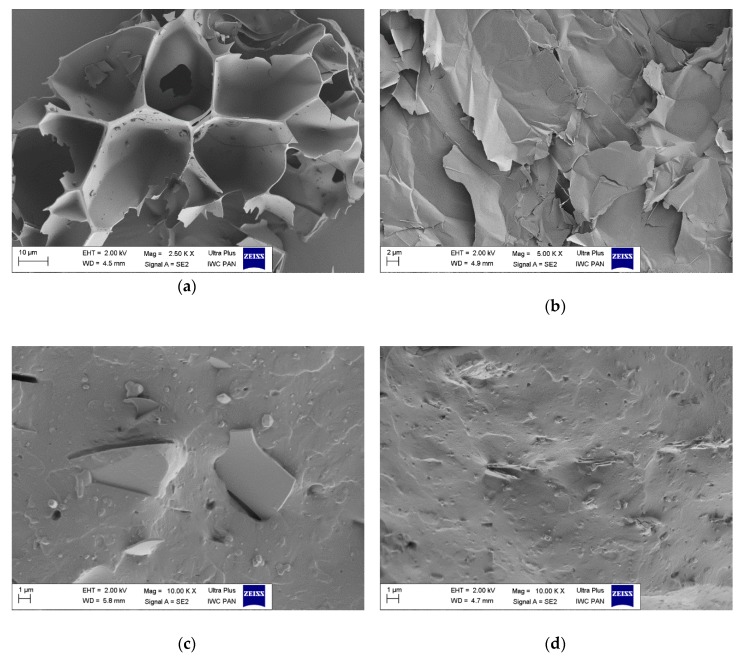
SEM microphotographs of: (**a**) perlite; (**b**) vermiculite; (**c**) EPM composite filled with 10 phr of perlite; (**d**) EPM composite filled with 10 phr of vermiculite.

Figure 8a–d shows SEM micrographs of the filler particles and EPM composites containing 10 phr of each filler. Figure 8a depicts the microstructure of the PT raw granules, characterized by fragmented surfaces with irregular small shreds and a highly porous microstructure containing mainly open pores. The VMT granules have different sizes, in the range of a several microns, with a platelet structure. Figure 8c,d shows cross-sections of the EPM/PT and EPM/VMT composites. It appears that both composites consist of a mixture of individual platelets of VMT or PT and some stacks of platelets.

## 4. Conclusions

This study investigated the effects of two natural minerals, perlite (PT) and vermiculite (VMT), on the properties of ethylene–propylene rubber composites. Three different concentrations of the fillers were used (5, 10 and 20 phr). TFSI-based imidazolium ionic liquids were added to improve filler–polymer compatibility. Comprehensive tests revealed that the studied minerals had a considerable effect on the curing and mechanical properties of the EPM elastomer. The application of PT contributed to improve the torque moment, crosslink density, and tensile strength of the compounds. On the other hand, VMT had a more pronounced impact on the tear resistance and barrier performance of the EPM systems. The varied effects of the studied fillers on the mechanical and barrier properties of the EPM compounds probably resulted from their different structures. It was found that ionic liquids had a catalytic effect on the crosslinking process. Importantly, TGA and CCT analyses revealed that the studied minerals and ionic liquids had a strong effect on the thermal and flame-retardant properties of the EPM compounds, especially in the case of EPM/PT systems. This can be explained by the inhibited rate of degradation in the initial stage and the very high thermal insulation properties of the studied minerals. Therefore, both PT and VMT can be considered for use in the preparation of novel and inexpensive elastomeric materials with improved flame retardancy and gas barrier stability, suitable for important and practical applications in rubber technology.

## Figures and Tables

**Table 1:** Composition of EPM-filled mixtures (in phr—parts per hundred parts of rubber).

Compound	Neat EPM	DCP	TTT	Perlite	Vermiculite	AMIMTFSI	BMIMTFSI
EPM	100	1	0.25	0	0	0	0
EPM/5PT	100	1	0.25	5	0	0	0
EPM/10PT	100	1	0.25	10	0	0	0
EPM/20PT	100	1	0.25	20	0	0	0
EPM/20PT/AMIMTFSI	100	1	0.25	20	0	2.5	0
EPM/20PT/BMIMTFSI	100	1	0.25	20	0	0	2.5
EPM/5VMT	100	1	0.25	0	5	0	0
EPM/10VMT	100	1	0.25	0	10	0	0
EPM/20VMT	100	1	0.25	0	20	0	0
EPM/20VMT/AMIMTFSI	100	1	0.25	0	20	2.5	0
EPM/20VMT/BMIMTFSI	100	1	0.25	0	20	0	2.5

**Table 2:** Rheometric parameters and crosslink density values of EPM-filled compounds.

Compound	M_min_ (dNm)	ΔM (dNm)	t_02_ (min)	t_90_ (min)	υ_e_ (mol/cm^3^)
EPM	0.81	4.80	1.07	18.84	3.4
EPM/5PT	0.91	5.41	1.09	17.29	4.5
EPM/10PT	0.98	6.08	1.03	17.20	5.3
EPM/20PT	1.14	7.19	0.91	15.76	5.9
EPM/20PT/AMIMTFSI	0.96	5.37	0.83	14.16	5.0
EPM/20PT/BMIMTFSI	0.97	5.42	0.90	15.28	4.8
EPM/5VMT	0.87	4.83	1.16	14.76	3.5
EPM/10VMT	0.93	4.86	1.03	13.29	3.6
EPM/20VMT	0.99	4.45	1.06	12.76	2.8
EPM/20VMT/AMIMTFSI	0.96	5.90	1.01	11.57	4.3
EPM/20VMT/BMIMTFSI	0.96	5.62	1.02	11.58	4.9

M_min_—minimum torque moment, ∆M—increment of torque, t_02_—scorch time, t_90_—optimum curing time, υ_e_—crosslink density.

**Table 3:** Thermogravimetric data for EPM composites.

Compound	T_5_ (°C)	T_50_ (°C)	T_RMAX1_ (°C)	T_RMAX2_ (°C)	dm/dt_1_ (%/min)	dm/dt_2_ (%/min)	P_600_ (%)
EPM	298	423	350	442	4.85	5.36	18.82
EPM/5PT	271	400	300	403	1.95	23.53	1.17
EPM/10PT	272	405	305	401	1.38	17.3	6.63
EPM/20PT	278	410	270	407	1.50	34.76	12.61
EPM/5VMT	288	405	292	398	1.33	30.34	10.42
EPM/10VMT	262	404	260	402	1.24	39.71	4.18
EPM/20VMT	296	410	261	403	0.93	20.16	15.65

T_5, 50_—temperature of sample 5% and 50% mass loss, T_RMAX_—temperature for maximum rate of thermal decomposition of tested vulcanizates, P_600_—residue after thermal decomposition of the composites.

**Table 4:** Flammability parameters of EPM composites.

Compound	TTI (s)	HRR (kW/m^2^)	HRR_max_ (kW/m^2^)	THR (MJ/m^2^)	EHC (MJ/kg)	EHC_max_ (MJ/kg)	MLR (g/m^2^∙s)
EPM	70	202.7	576.0	92.9	51.0	79.0	0.206
EPM/5PT	68	204.1	445.9	57.7	31.8	77.5	0.257
EPM/10PT	70	195.5	390.5	49.8	38.1	79.2	0.212
EPM/20PT	90	66.34	189.7	16.6	10.4	64.2	0.224
EPM/20PT/AMIMTFSI	120	61.23	179.3	17.0	8.9	62.1	0.200
EPM/20PT/BMIMTFSI	110	63.52	170.2	16.2	9.3	60.2	0.265
EPM/5VMT	70	185.3	560.1	70.1	46.6	72.1	0.211
EPM/10VMT	73	171.8	529.6	47.9	75.8	78.2	0.014
EPM/20VMT	80	177.3	435.8	53.9	29.8	77.2	0.023
EPM/20VMT/AMIMTFSI	105	145.7	401.6	51.5	26.9	74.2	0.245
EPM/20VMT/BMIMTFSI	75	120.5	380.5	49.7	21.3	69.5	0.213

TTI—time to ignition; HRR—heat release rate; HRR_max_—max heat release rate; THR—total heat release; EHC—effective heat of combustion; EHC_max_—max effective heat of combustion; MLR—mass loss rate.
